# Influenza vaccine compatibility among hospitalized patients during and after the COVID-19 pandemic

**DOI:** 10.3389/fmicb.2023.1296179

**Published:** 2024-01-23

**Authors:** Ilana S. Fratty, Menucha Jurkowicz, Neta Zuckerman, Ital Nemet, Nofar Atari, Limor Kliker, Lea Gur-Arie, Alina Rosenberg, Aharona Glatman-Freedman, Yaniv Lustig, Michal Mandelboim

**Affiliations:** ^1^Central Virology Laboratory, Public Health Services, Ministry of Health and Sheba Medical Center, Ramat-Gan, Israel; ^2^The Israel Center for Disease Control, Israel Ministry of Health, Ramat-Gan, Israel; ^3^Faculty of Medicine, Department of Epidemiology and Preventive Medicine, Tel-Aviv University, Tel-Aviv, Israel

**Keywords:** influenza A, influenza B, vaccine, hemagglutinin, SARS-CoV-2

## Abstract

**Introduction:**

Following the significant decrease in SARS-CoV-2 cases worldwide, Israel, as well as other countries, have again been faced with a rise in seasonal influenza. This study compared circulating influenza A and B in hospitalized patients in Israel with the influenza strains in the vaccine following the 2021–2022 winter season which was dominated by the omicron variant.

**Methods:**

Nasopharyngeal samples of 16,325 patients were examined for the detection of influenza A(H1N1)pdm09, influenza A(H1N1)pdm09 and influenza B. Phylogenetic trees of hemagglutinin were then prepared using sanger sequencing. Vaccine immunogenicity was also performed using the hemagglutination inhibition test.

**Results:**

Of the 16,325 nasopharyngeal samples collected from hospitalized patients between September 2021 (Week 40) and April 2023 (Week 15), 7.5% were found to be positive for influenza. Phylogenetic analyses show that in the 2021–2022 winter season, the leading virus subtype was influenza A(H3N2), belonging to clade 3C.2a1b.2a.2. However, the following winter season was dominated by influenza A(H1N1)pdm09, which belongs to clade 6B.aA.5a.2. The circulating influenza A(H1N1)pdm09 strain showed a shift from the vaccine strain, while the co-circulating influenza A(H3N2) and influenza B strains were similar to those of the vaccine. Antigenic analysis coincided with the sequence analysis.

**Discussion:**

Influenza prevalence during 2022–2023 returned to typical levels as seen prior to the emergence of SARS-CoV-2, which may suggest a gradual viral adaptation to SARS-CoV-2 variants. Domination of influenza A(H1N1)pdm09 was observed uniquely in Israel compared to Europe and USA and phylogenetic and antigenic analysis showed lower recognition of the vaccine with the circulating influenza A(H1N1)pdm09 in Israel compared to the vaccine.

## Introduction

In temperate and continental climates, influenza outbreaks occur every year, most commonly in the winter when humidity and temperature levels are low. In tropical and sub-tropical climates, it can occur year-round (Uyeki et al., 2022). Influenza infection is associated with respiratory and non-respiratory complications such as pneumonia, bacterial co-infection, and cardiac complications that can lead to hospitalization and morbidity. An estimated 300,000 influenza-related deaths occur annually worldwide (Petrova and Russell, 2018).

Annual vaccination is considered the most effective approach to preventing influenza (Uyeki et al., 2022). The composition of the annual vaccine is determined by the World Health Organization (WHO) based on worldwide surveillance data (WHO, 2023a). However, studies show low vaccine effectiveness (VE), especially in vaccines containing the influenza A(H3N2) strain, which drifts and adapts new mutations (Huddleston et al., 2020). Vaccine preparation also poses significant challenges. Generally, influenza vaccines are produced using an egg-based manufacturing process. However, the virus can acquire mutations and drift during egg adaptation, which further contributes to low VE. Hence, the vaccine composition is evaluated every year and updated according to the current circulating strains (WHO, 2023a,b).

Prior to the COVID-19 pandemic, influenza seasons in temperate areas were generally dominated by a single strain, with other influenza strains circulating at lower levels. As SARS-CoV-2 cases decreased in Israel in September 2021, influenza cases increased and were dominated by influenza A(H3N2), with no other strains circulating, while in the United States and Europe, other influenza strains were circulating [Grohskopf et al., 2016; Centers for Disease Control and Prevention (CDC), 2023; Europe Centers for Disease Control and Prevention (ECDC), 2023; Price et al., 2023]. In this study, we characterized the influenza seasons 2021–2022 and 2022–2023, during which SARS-CoV-2 circulation transitioned from the pandemic to the post-pandemic phase, and examined the compatibility of the influenza vaccine with circulating strains in Israel as compared to the United States and Europe.

## Methods

### Patients and samples

Nasopharyngeal samples of patients hospitalized at Sheba Medical Center (SMC), the largest tertiary medical center in Israel (1,619 hospital beds), were received at the Central Virology Laboratory of the Ministry of Health located on the premises of SMC. In total, 16,325 samples were tested for the influenza virus between week 40 of 2021 (which started October 2, 2021) and week 15 of 2023 (which started April 9, 2023). The distribution of samples by patients’ age, sex, and critical hospitalization is presented in the Supplementary Tables S1, S2.

### Viral RNA extraction and real-time PCR

Viral RNA was extracted using the STARMag Viral DNA/RNA 200C universal kit (Seegene Inc., Korea). Reverse transcription and RT-PCR assays were performed using the AllplexTM RV essential assay (Seegene Inc., Korea) in a CFX real-time PCR system (BIO-RAD, United States; Folgueira et al., 2019). Influenza typing for A(H1N1) and A(H3N2) was performed as previously described (Meningher et al., 2014; Pando et al., 2021). Briefly, the master mix was prepared with the SensiFAST kit (Meridian Bioscience, United Kingdom) using specific primers for influenza A(H3N2) and influenza A (H1N1pdm) that are listed in Supplementary Table S3 (Pando et al., 2021). The lineage of influenza B was determined by RT-PCR The qRT-PCR assay was performed on a CFX real-time PCR system (BIO-RAD, United States). The influenza B lineage was determined by Sanger sequencing of the hemagglutinin, as described below.

### Sequencing and genomic analysis

Hemagglutinins (HA) from 77 laboratory-confirmed influenza viruses were randomly selected for sequencing. Sequencing was performed using the Sanger method and analyzed using the Sequencher software (Gene Codes Corporation, United States). In brief, qRT-PCR was performed on positive influenza samples using the One-Step RT-PCR kit (Qiagen, Germany), separated on a 2% agarose gel, and visualized by agarose gel electrophoresis. The primers that were used for the sequencing are listed in Supplementary Table S4. PCR products were purified with the EPPiC Fast enzyme (A&A Biotechnology, Poland). The DNA templates were sequenced using the BigDye Terminator v1.1 kit on an ABI Prism 3100 automated sequencer (Applied Biosystems, United States). Phylogenetic analysis of each influenza strain was performed by Geneious software (Dotmatics) using the neighbor-joining method. All ID numbers of sequences that were retrieved for phylogenetic analysis are listed in Supplementary Index 2.

### Vaccine immunogenicity

To analyze how well the vaccine recognized currently circulating viruses, a hemagglutination inhibition (HI) test was performed. The HI test was performed by the WHO Collaborating Center, London, according to the standard WHO procedure using ferret post-infection antisera raised against the vaccine strains (WHO, 1997).

### Protein illustration

The HA protein structure was visualized using PyMOL software (Schrodinger, United States). Influenza sequences were retrieved from the EpiFluTM database of the Global Initiative on Sharing All Influenza Data (GISAID) (GISAID, 2023).

### International comparisons

The predominant influenza virus types and subtypes detected among SMC hospitalized patients were compared to those of other regions in the Northern Hemisphere (WHO, 2023c).

### SARS-CoV-2 analysis

The data on new SARS-CoV-2 cases in Israel are published daily by the Israel Ministry of Health (IMoH) and follow the rate of COVID-19 since February 2020 (WHO, 2023d). The data on SARS-CoV-2 variants are attributed to the World Consortium of SARS-CoV-2 sequences, which updates the variants of concern daily (Nextstrain, 2023).

## Results

### Influenza virus presence among SMC hospitalized patients

**Figure 1:**
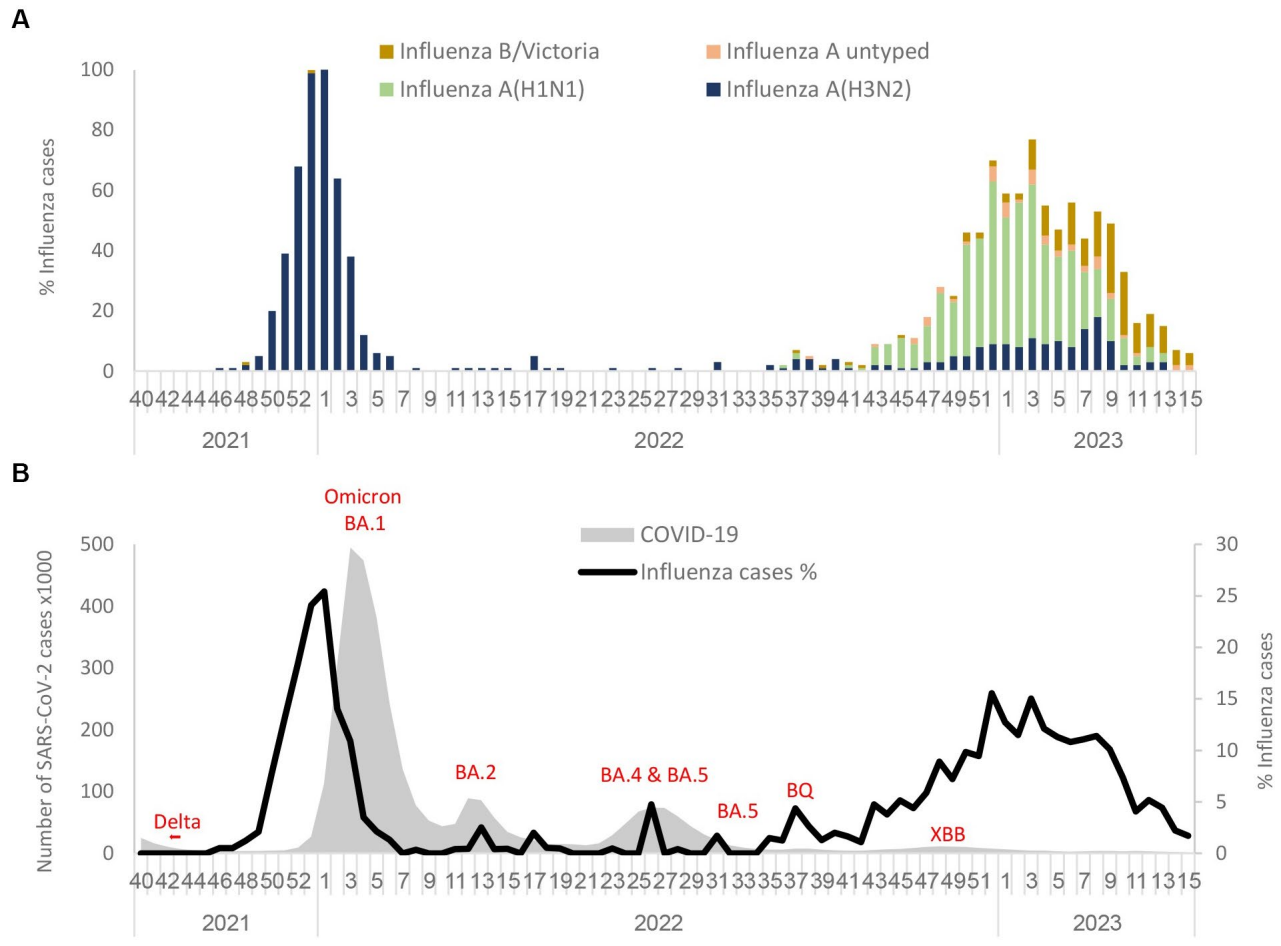
Circulation of influenza subtypes **(A)** and SARS-CoV2 **(B)** from 2021 to April 2023 in Israel. Influenza A(H1N1)pdm09, influenza A(H3N2), influenza B (Victoria), and influenza A untyped were collected and analyzed in the period of 2021 to April 2023 in SMC hospitalized patients **(A)**. Number of SARS-CoV-2 detected in Israel from 2021 to 2023 along with the percentage of influenza rate in SMC hospitalized patients **(B)**.

During the 2021–2022 winter season, an increased percentage of influenza viruses was detected in November 2021 among samples from hospitalized patients that were abruptly interrupted by the arrival of the omicron variants. As shown in Figure 1A, only the influenza A(H3N2) subtype was detected in the winter season of 2021–2022. However, in the 2022–2023 winter season, influenza A(H1N1)pdm09 cases emerged with a lower percentage of influenza A(H3N2) and influenza B/Victoria lineages. The predominant strain in the 2022–2023 winter season was influenza A(H1N1)pdm09. At the end of the influenza season (March–April 2023), the percentage of influenza A cases decreased, while the percentage of influenza B-positives remained stable. Figure 1B represents the percentage of influenza rate alongside SARS-CoV-2 variants from September 2021 to March–April 2023. With the introduction of the omicron BA.1 variant, the influenza rate decreased immediately in January 2022. However, BA.2, BA.4, BA.5, BQ, and XBB variants that were present in Israel during 2022 and 2023 were not accompanied by a decrease in the influenza rate. Additionally, a cross-checking analysis of double infection by SARS-CoV-2 and influenza was also performed and showed only five cases of double infection (0.08%). The comparison of critical cases between patients with positive influenza A(H1N1)pdm09 vs. influenza A(H3N2) showed no significant difference (Supplementary Table S2).

### International comparisons

During the 2021–2022 season, influenza A(H3N2) was the predominant influenza virus in Central Asia, Europe, and North America in addition to Israel (Table 1). In the 2022–2023 season, influenza A(H1N1)pdm09 was predominant in Central Asia and Eastern Europe in addition to Israel. Influenza A(H3N2) was predominant in Southern Asia, Southwest Europe, and North America. Influenza B(Victoria) was predominant in Eastern Asia during both seasons.

**Table 1:** Predominant influenza strains in Asia, Israel, Europe, and North America.

	2021–2022	2022–2023
Central Asia	A(H3N2)	A(H1N1)pdm09
Eastern Asia	B (Victoria)	B (Victoria)
Southern Asia	A(H3N2)	A(H3N2)
Western Asia	A(H3N2)	A(H1N1)pdm09 + A(H3N2)
Israel	A(H3N2)	A(H1N1)pdm09
Northern Europe	A(H3N2)	A(H1N1)pdm09 + A(H3N2)
Eastern Europe	A(H3N2)	A(H1N1)pdm09
South-West Europe	A(H3N2)	A(H3N2)
North America	A(H3N2)	A(H3N2)

### Comparison of the influenza vaccine HA to influenza virus HA detected among SMC hospitalized patients

**Figure 2:**
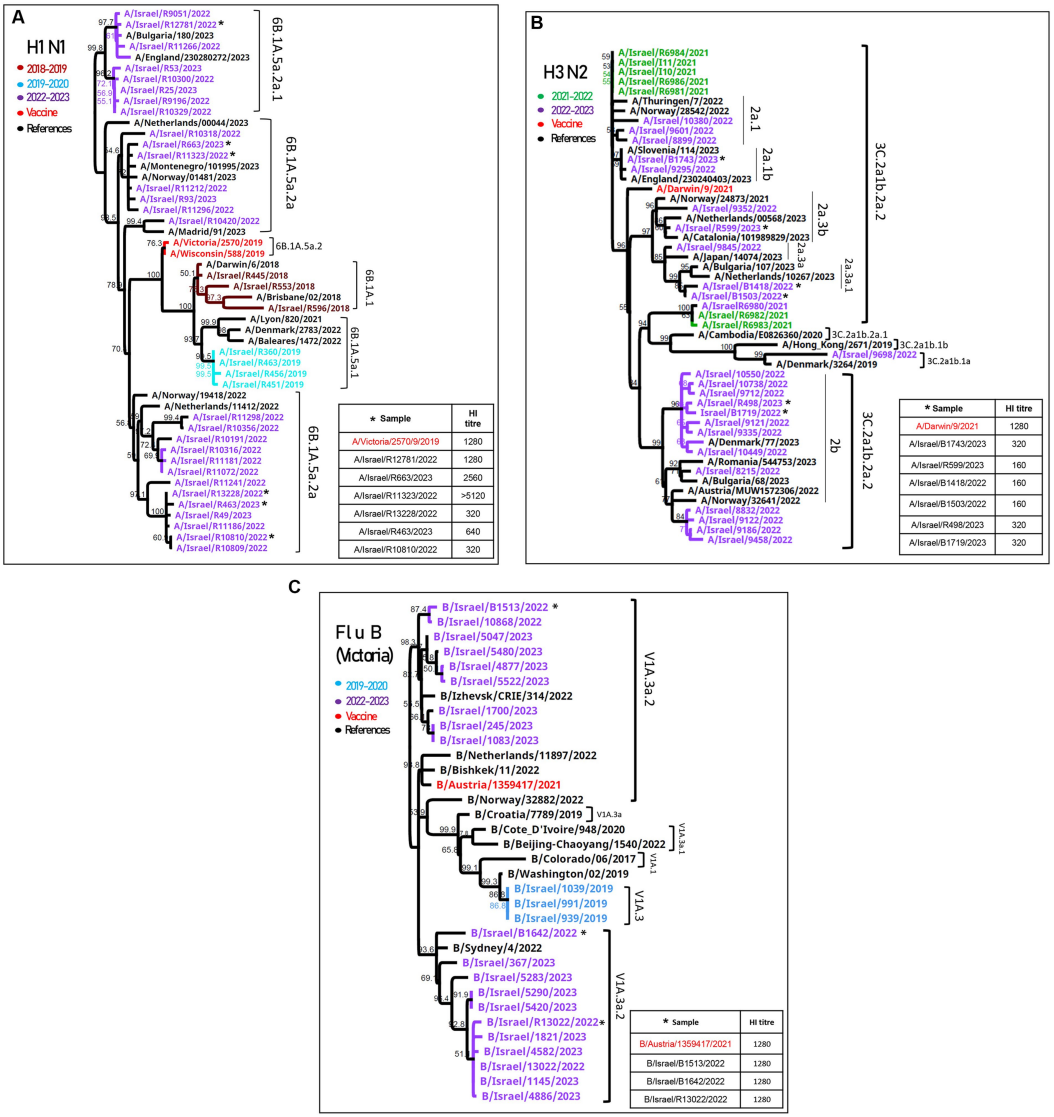
Phylogenetic and antigenic analyses of influenza A(H1N1)pdm09, influenza A(H3N2), and influenza B. Phylogenetic trees of patients’ samples positive for influenza in SMC hospitalized patients in the 2022–2023 season of (H1N1)pdm09 **(A)**, influenza (H3N2) **(B)**, and influenza B/Victoria **(C)**. Antigenic analysis of each strain is presented in the right corner of each figure. The samples that were tested for hemagglutination inhibition (HI) are indicated with an asterisk.

Influenza A(H1N1)pdm09 detected during the 2022–2023 season were of the 6B.1A.5a.2a and 6B.1A.5a.2a.1 clades (Figure 2A). The vaccine strains (A/Wisconsin/588/2019 and A/Victoria/2570/2019) belong to 6B.1A.5a.2, which are located in a different branch from the samples detected in Israel, as shown in Figure 2A. Antigenic analysis is also shown in Figure 2A, in the right corner. The samples that were analyzed are indicated in the phylogenetic tree with an asterisk. Of the six samples, three (A/Israel/R12781/2022, A/Israel/R663/2023, and A/Israel/R11323/2022) were located in the upper branches and showed high hemagglutination inhibition (HI) titer (>1,280), while the samples A/Israel/R13228/2022, A/Israel/R463/2023, and A/Israel/R10810/2022 show low HI titer (<640) and were located in a different branch.

The HA of influenza A(H3N2) samples from both seasons (2021–2022 and 2022–2023) were found to belong to the 3C.2a1b.2a.2 clade, adjacent to the vaccine clade (A/Drawin/9/2021) (Figure 2B). The antigenic analysis of six more samples shown in the right corner of Figure 2B had a low HI titer compared to the vaccine.

The influenza B/Victoria sequences were of the V1a.3a.2 lineage, similar to the vaccine clade (B/Austria/1359417/2021), and the antigenic analysis showed a similar HI titer compared to the vaccine (Figure 2C).

**Figure 3:**
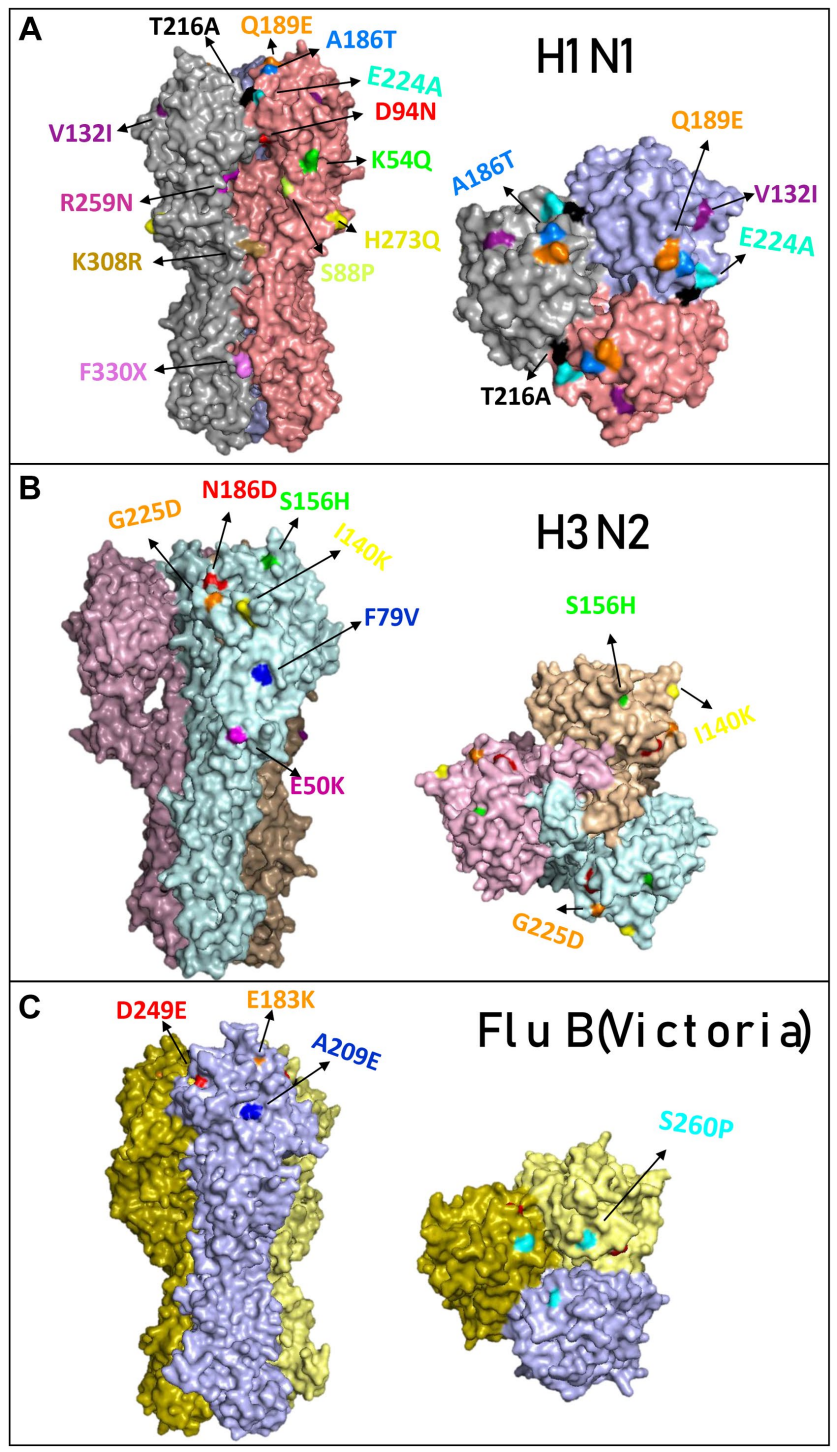
Overview of the hemagglutinin amino acid substitutions of the influenza isolated from hospitalized patients compared to the vaccine. Modeling of hemagglutinin (HA) and its amino acid substitutions compared to the vaccine. The samples were compared to the vaccines for influenza A(H1N1)pdm09 (compared with sample A/Israel/R13228/2022) **(A)**, influenza (H3N2) (compared with sample A/Israel/R8215/2022) **(B)**, and influenza B/Victoria (compared with sample B/Israel/R13022/2022) **(C)**. Mutations found in the 2022–2023 season are colored differently.

A comparison of the structure models of the vaccine homo-trimer (A/Wisconsin/588/2019) HA of influenza can be seen in Figure 2C. The main mutations that were detected in Israel are highlighted by different colors (Figure 3A). The mutations found for influenza A(H1N1)pdm09 were K54Q, S88P, D94N, V132I, A186T, Q189E, T216A, E224A, R259N H273Q, K308R, and F330X. A list of all the amino acid substitutions is presented in Supplementary Index 3. The HA modeling of influenza A(H3N2) in Figure 3B emphasizes the mutations detected in the positive samples of influenza A(H3N2) in the 2022–2023 season. The mutations found in Israel were E50K, F79V, I140K, S156H, N186D, and G225D. The influenza B mutations identified in Israel were E183K, A209E, D249E, and S260P (Figure 3C).

## Discussion

Analysis of influenza incidence prior to and during the COVID-19 pandemic identified a significantly lower number of cases alongside the emergence of the SARS-CoV-2 alpha and delta variants, as seen in other reports (Olsen et al., 2021; Lauring et al., 2022). As with other SARS-CoV-2 variants, the emergence of the omicron variant in December 2021 was followed by a dramatic reduction in influenza cases among hospitalized patients in Israel (Figure 1). The results shown in this study (Figure 1) demonstrate that during the 2022–2023 season, influenza activity among hospitalized patients and the epidemic curve were similar to those of the pre-pandemic seasons. This “typical” influenza pattern among hospitalized patients occurred, while the circulation of the SARS-CoV-2 variants was low throughout the season (WHO, 2023c,d). The possibility of dual infection of SARS-CoV-2 and influenza was also examined in this study, as dual infection may lead to severe cases and increased mortality (Bai et al., 2021; Tang et al., 2022). However, a very low percentage of such cases were found (0.08%), presumably as a result of SARS-CoV-2 circulation at that time.

In the 2021–2022 season, the influenza virus detected among hospitalized patients belonged to influenza A(H3N2), clade 3C.2a1b.2a.2. This clade was the predominant influenza virus throughout the world in the 2021–2022 season (WHO, 2023a). Subsequently, in 2022–2023, clade 3C.2a1b.2a.2 was still circulating in the world; however, influenza A(H3N2) was less frequent in hospitalized patients in Israel. The predominant virus in the 2022–2023 season was influenza (H1N1)pdm09, which was not detected in 2021–2022, while in Southern Asia, most of Europe, and North America, influenza A(H3N2) predominated [Centers for Disease Control and Prevention (CDC), 2023; Europe Centers for Disease Control and Prevention (ECDC), 2023]. Influenza B/Victoria was also detected but less frequently in hospitalized patients, and its positive rate remained stable at the end of the 2022–2023 season, while influenza A decreased, corresponding with its typical end-of-the-season upticks (Uyeki et al., 2022). Influenza B/Yamagata was not detected among hospitalized patients in Israel, similar to global findings during the COVID-19 pandemic (Koel et al., 2013).

The phylogenetic analysis of the 2022–2023 influenza A(H1N1)pdm09 strain found a lack of compatibility between the vaccine and the circulating influenza A(H1N1)pdm09 viruses among hospitalized patients in Israel. While the vaccine virus belongs to 6B.1A.5a.1, the influenza A(H1N1)pdm09 virus detected in patients evolved into two clades, 6B.1A.5a.2a and 6B.1A.5a.2a.1, similar to influenza A(H1N1)pdm09 viruses detected in Europe. This result coincides with the immunogenicity experiments, which showed a low HI titer that indicates a lack of compatibility between the circulating strain and the vaccine. Reduced compatibility between the vaccine and the circulating influenza viruses is attributed to the amino acid substitutions in hemagglutinin positions K54Q, A186T, Q189E, E224A, R259K, and K308R detected in hospitalized patients in Israel (Supplementary Index 3). Mutations in positions 186 and 189 have been found to belong to the patches responsible for major antigenic change (Koel et al., 2013; Kratsch et al., 2016). Influenza A(H3N2) also evolved into smaller clades from the 3C.2a1b.2a.2 clade, but reports from the WHO showed that compatibility with the influenza A(H3N2) vaccine remained (WHO, 2023a).

Despite the match between the vaccine and the circulating influenza A(H3N2), the United States was still burdened by more influenza A(H3N2) infections, possibly due to the low vaccine coverage in the population (Uyeki et al., 2022). In Israel in 2022–2023, vaccination rates were relatively low (17.9%). Nevertheless, the influenza A(H3N2) infection rate was lower in Israel compared with the United States and Europe, which may have been due to immunity gained in the 2021–2022 winter season when influenza A(H3N2) circulated. In contrast, influenza A(H1N1)pdm09 last circulated in Israel in the winter season of 2019–2020, which may explain its dominance in Israel in 2022–2023 (Fratty et al., 2022; Omer et al., 2022; Malosh et al., 2023). Overall, the prevalence of influenza A strains tends to shift each year between influenza A(H1N1)pdm09 and influenza A(H3N2), as seen prior to the emergence of SARS-CoV-2 in Israel [Europe Centers for Disease Control and Prevention (ECDC), 2023].

For each coming winter season, a composition for the influenza vaccine is recommended by the WHO after examining the recent circulating subtypes (WHO, 2023a). Since the influenza A(H1N1)pdm09 included in the vaccine was incompatible in 2022–2023, the WHO recommended the inclusion of influenza with stronger clade-specific recognition (WHO, 2023a,b). Hence, we suggest removing the B/Yamagata component from the vaccine, using a higher antigen content in targeted populations (such as older adults), and using cell culture for propagation, a procedure that introduces fewer molecular changes (Uyeki et al., 2022). Alternatively, other technologies, such as mRNA technology, which was proven to be a game-changer during the COVID-19 pandemic, can be used.

In conclusion, higher rates of influenza activity were measured in the 2022–2023 season as compared with the 2021–2022 season. The influenza strain that dominated the 2022–2023 season was unique in Israel compared to Europe and the United States. The majority of influenza samples in hospitalized patients in Israel in 2022–2023 belonged to influenza A(H1N1)pdm09, but influenza A(H3N2) and influenza B/Victoria were also detected. Phylogenetic and antigenic analyses of two out of the three influenza types detected in Israel showed a resemblance to the vaccine recommended by the WHO, with the exception of influenza A(H1N1)pdm09.

## Data availability statement

The datasets presented in this study can be found in online repositories. The names of the repository/repositories and accession number(s) can be found in the article/Supplementary material.

## Ethics statement

The studies involving humans were approved by the institutional review board (IRB) of the Sheba Medical Center (Helsinki Number 7688-20-SMC). The studies were conducted in accordance with the local legislation and institutional requirements. This study was a retrospective using anonymized data, so did not require written informed consent.

## Author contributions

IF: Investigation, Writing – original draft, Formal analysis. MJ: Writing – review & editing. NZ: Writing – review & editing. IN: Investigation, Writing – review & editing. NA: Investigation, Writing – review & editing. LK: Investigation, Writing – review & editing. LG-A: Writing – review & editing, Formal analysis. AR: Writing – review & editing, Formal analysis. AG-F: Supervision, Writing – review & editing. YL: Supervision, Writing – review & editing. MM: Supervision, Writing – review & editing.
