# Supplementary material for: Influenza vaccine compatibility among hospitalized patients during and after the COVID-19 pandemic
Source: Front Microbiol. 2024 Jan 23;14:1296179. doi: 10.3389/fmicb.2023.1296179 (PMC10844098; doi:10.3389/fmicb.2023.1296179)
Supplement: Supplementary file 1 [file Table_1.DOCX]

Supplementary data

Table S1. Distribution of samples tested for influenza by age group and sex

*

*

*Week 40 in 2021 to week 15 in 2022. ** Week 40 in 2022 to week 15 in 2023

|  | 2022-2023** | | |  | 2021-2022* | | | Winter season | |
| --- | --- | --- | --- | --- | --- | --- | --- | --- | --- |
| P value | Influenza- positive samples | | Sample tested |  | Influenza- positive samples | | Sample tested |  |  |
|  | % | n | n |  | % | n | n |  |  |
| 0.28 | 7.1 | 93 | 1301 |  | 4.6 | 32 | 695 | 0-2 | Age group (years) |
| 0.09 | 18.2 | 98 | 539 |  | 10.2 | 32 | 315 | 3-9 |  |
| 0.38 | 14.6 | 29 | 199 |  | 7.7 | 7 | 91 | 10-14 |  |
| 0.07 | 10.7 | 43 | 402 |  | 71.1 | 43 | 252 | 15-24 |  |
| 0.82 | 14.6 | 133 | 909 |  | 14.3 | 78 | 544 | 25-44 |  |
| 0.04 | 8.8 | 152 | 1723 |  | 6.6 | 65 | 987 | 45-65 |  |
| 0.84 | 6.5 | 335 | 5124 |  | 5.7 | 180 | 3176 | >65 |  |
|  | 7.3 | 3 | 41 |  | 11.1 | 3 | 27 | Unknown |  |
|  | 8.1 | 431 | 5309 |  | 6.6 | 217 | 3256 | Male | Sex |
|  | 9.3 | 452 | 4859 |  | 7.8 | 221 | 2811 | Female |  |
|  | 4.2 | 3 | 70 |  | 1 | 2 | 20 | Unknown |  |
| 0.6 | 8.6 | 886 | 10238 |  | 7.2 | 440 | 6087 | Total |  |

*Week 40 in 2021 to week 15 in 2022 ** Week 40 in 2022 to week 15 in 2023

Table S2. Critical hospitalization of patients with positive influenza detection

| ≥18 | | | <18 | | | | Age |  |
| --- | --- | --- | --- | --- | --- | --- | --- | --- |
| P value | H3N2 | H1N1 | P value | H3N2 | H1N1 | | Influenza type |  |
| 0.38 | 6.2% | 5.1% | 0.66 | 6.4% | 6.5% | Intensive care unit (ICU) | | |
|  | 8.0% | 10.5% |  | 16.5% | 13.1% | Oncologic | |  |
|  | 85.8% | 84.4% |  | 77.1% | 80.4% | | All departments |  |

Table S3. List of primers that were used for Real-Time PCR for influenza A(H1N1)pdm09 , influenza A(H3N2) and influenza B/Victoria detection.

| **Influenza type** | **Primer** | **Sequence 5'-3'** |
| --- | --- | --- |
| H1N1pdm09 | Forward | CATTTGAAAGGTTTGAGATATTCCC |
| H1N1pdm09 | Reverse | ATGCTGCCGTTACACCTTTGT |
| H1N1pdm09 | Probe | Cy5-ACAAGTTCATGGCCCAATVATGACTCG-BHQ |
| H3N2 | Forward | AATGGTTGGGAGGGAATG |
| H3N2 | Reverse | TTGAGTGCTTTTRAGAACTG |
| H3N2 | Probe | Vic-TTGGTAGGGTTTCAGGCATCA-TAMARA |
| B/Victoria | Forward | GATCTGGACGTAGCCTTGGG |
| B/Victoria | Reverse | TAACAGGTCTGACTTCATGGAG |
| B/Victoria | Probe | Cy5-TTCCCCGTGCATTTTG-BHQ |

Table S4. List of primers that were used for sequencing of Influenza A and B.

| **Influenza type** | **Gene** | **Position** | **Sequence 5'-3'** |
| --- | --- | --- | --- |
| H1N1pdm09 | Hemagglutinin | 1-943 | Fw-TGT AAA ACG ACG GCC AGT ATA CGA CTA GCA AAA GCA GGG G  Rev- CAG GAA ACA GCT ATG ACC GAA AKG GGA GRC TGG TGT TTA |
| H1N1pdm09 | Hemagglutinin | 736-1778 | Fw-TGT AAA ACG ACG GCC AGT ACR TGT TAC CCW GGR GAT TTC A  Rev- CAG GAA ACA GCT ATG ACC GTG TCA GTA GAA ACA AGG GTG TTT |
| H3N2 | Hemagglutinin | 6-1075 | Fw- AAGCAGGGGATAATTCTATTAACC  Rev- AACCGTACCAACCRTCCACCATTC |
| H3N2 | Hemagglutinin | 567-1705 | Fw- CTGAACGTGACTATGCCAAACAA  Rev-TCAAATGCAAATGTTTGC |
| B/Victoria | Hemagglutinin | 1-1166 | Fw-AATATCCACAAAATGAAGGCAATA  Rev- ATCATTCCTTCCCATCCTCCTTCT |
| B/Victoria | Hemagglutinin | 990-1793 | Fw-ACAAAAGCAAGCCTTACTACA  Rev- TTATAGACAGATGGAGC |
