# Supplementary material for: Influenza vaccine compatibility among hospitalized patients during and after the COVID-19 pandemic
Source: Front Microbiol. 2024 Jan 23;14:1296179. doi: 10.3389/fmicb.2023.1296179 (PMC10844098; doi:10.3389/fmicb.2023.1296179)
Supplement: Supplementary file 3 [file Table_3.DOCX]

**Supplementary 2**

Comparison of the amino acids of the vaccine (A/Wisconsin/588/2019) and the samples that were detected positive for influenza (H1N1)09pdm

A/Wisconsin/588/2019 MKAILVVMLYTFTTANADTLCIGYHANNSTDTVDTVLEKNVTVTHSVNLLEDKHNGKLCK

R12781/2022 MKAILVVMLYTFTTANADTLCIGYHANNSTDTVDTVLEKNVTVTHSVNLLEDKHNGKLCK

R9051/2022 MKAILVVMLYTFTTANADTLCIGYHANNSTDTVDTVLEKNVTVTHSVNLLEDKHNGKLCK

R11266/2022 MKAILVVMLYTFTTANADTLCIGYHANNSTDTVDTVLEKNVTVTHSVNLLEDKHNGKLCK

R10300/2022 MKAILVVMLYTFTTANADTLCIGYHANNSTDTVDTVLEKNVTVTHSVNLLEDKHNGKLCK

R9196/2022 MKAILVVMLYTFTTANADTLCIGYHANNSTDTVDTVLEKNVTVTHSVNLLEDKHNGKLCK

R10329/2022 MKAILVVMLYTFTTANADTLCIGYHANNSTDTVDTVLEKNVTVTHSVNLLEDKHNGKLCK

R25/2023 MKAILVVMLYTFTTANADTLCIGYHANNSTDTVDTVLEKNVTVTHSVNLLEDKHNGKLCK

R53/2023 MKAILVVMLYTFTTANADTLCIGYHANNSTDTVDTVLEKNVTVTHSVNLLEDKHNGKLCK

R10810/2022 MKAILVVMLYTFTTANADTLCIGYHANNSTDTVDTVLEKNVTVTHSVNLLEDKHNGKLCK

R463/2023 MKAILVVMLYTFTTANADTLCIGYHANNSTDTVDTVLEKNVTVTHSVNLLEDKHNGKLCK

R13228/2022 MKAILVVMLYTFTTANADTLCIGYHANNSTDTVDTVLEKNVTVTHSVNLLEDKHNGKLCK

R49/2023 MKAILVVMLYTFTTANADTLCIGYHANNSTDTVDTVLEKNVTVTHSVNLLEDKHNGKLCK

R11186/2022 MKAILVVMLYTFTTANADTLCIGYHANNSTDTVDTVLEKNVTVTHSVNLLEDKHNGKLCK

R10809/2022 MKAILVVMLYTFTTANADTLCIGYHANNSTDTVDTVLEKNVTVTHSVNLLEDKHNGKLCK

R11241/2022 MKAILVVMLYTFTTANADTLCIGYHANNSTDTVDTVLEKNVTVTHSVNLLEDKHNGKLCK

R10318/2022 MKAILVVMLYTFTTANADTLCIGYHANNSTDTVDTVLEKNVTVTHSVNLLEDKHNGKLCK

R10420/2022 MKAILVVMLYTFTTANADTLCIGYHANNSTDTVDTVLEKNVTVTHSVNLLEDKHNGKLCK

R663/2023 MKAILVVMLYTFTTANADTLCIGYHANNSTDTVDTVLEKNVTVTHSVNLLEDKHNGKLCK

R11323/2022 MKAILVVMLYTFTTANADTLCIGYHANNSTDTVDTVLEKNVTVTHSVNLLEDKHNGKLCK

R93/2023 MKAILVVMLYTFTTANADTLCIGYHANNSTDTVDTVLEKNVTVTHSVNLLEDKHNGKLCK

R11296/2022 MKAILVVMLYTFTTANADTLCIGYHANNSTDTVDTVLEKNVTVTHSVNLLEDKHNGKLCK

R11212/2022 MKAILVVMLYTFTTANADTLCIGYHANNSTDTVDTVLEKNVTVTHSVNLLEDKHNGKLCK

R11298/2022 MKAILVVMLYTFTTANADTLCIGYHANNSTDTVDTVLEKNVTVTHSVNLLEDKHNGKLCR

R10356/2022 MKAILVVMLYTFTTANADTLCIGYHANNSTDTVDTVLEKNVTVTHSVNLLEDKHNGKLCR

R10191/2022 MKAILIVMLYTFTTANADTLCIGYHANNSTDTVDTVLEKNVTVTHSVNLLEDKHNGKLCK

R10316/2022 MKAILVVMLYTFTTANADTLCIGYHANNSTDTVDTVLEKNVTVTHSVNLLEDKHNGKLCK

R11181/2022 MKAILVVMLYTFTTANADTLCIGYHANNSTDTVDTVLEKNVTVTHSVNLLEDKHNGKLCK

R11072/2022 MKAILVVMLYTFTTANADTLCIGYHANNSTDTVDTVLEKNVTVTHSVNLLEDKHNGKLCK

*****:*****************************************************.

A/Wisconsin/588/2019 LRGVAPLHLGKCNIAGWILGNPECESLSTARSWSYIVETSNSDNGTCYPGDFINYEELRE

R12781/2022 LRGVAPLHLGQCNIAGWILGNPECESLSTARSWSYIVETSNSDNGTCYPGDFINYEELRE

R9051/2022 LRGVAPLHLGQCNIAGWILGNPECESLSTARSWSYIVETSNSDNGTCYPGDFINYEELRE

R11266/2022 LRGVAPLHLGQCNIAGWILGNPECESLSTARSWSYIVETSNSDNGTCYPGDFINYEELRE

R10300/2022 LRGVAPLHLGQCNIAGWILGNPECESLSTARSWSYIVETSNSDNGTCYPGDFINYEELRE

R9196/2022 LRGVAPLHLGQCNIAGWILGNPECESLSTARSWSYIVETSNSDNGTCYPGDFINYEELRE

R10329/2022 LRGVAPLHLGQCNIAGWILGNPECESLSTARSWSYIVETSNSDNGTCYPGDFINYEELRE

R25/2023 LRGVAPLHLGQCNIAGWILGNPECESLSTARSWSYIVETSNSDNGTCYPGDFINYEELRE

R53/2023 LRGVAPLHLGQCNIAGWILGNPECESLSTARSWSYIVETSNSDNGTCYPGDFINYEELRE

R10810/2022 LRGVAPLHLGQCNIAGWILGNPECESLSTARSWSYIVETPNPDNGTCYPGNFINYEELRE

R463/2023 LRGVAPLHLGQCNIAGWILGNPECESLSTARSWSYIVETPNPDNGTCYPGNFINYEELRE

R13228/2022 LRGVAPLHLGQCNIAGWILGNPECESLSTARSWSYIVETPNPDNGTCYPGNFINYEELRE

R49/2023 LRGVAPLHLGQCNIAGWILGNPECESLSTARSWSYIVETPNPDNGTCYPGNFINYEELRE

R11186/2022 LRGVAPLHLGQCNIAGWILGNPECESLSTARSWSYIVETPNPDNGTCYPGNFINYEELRE

R10809/2022 LRGVAPLHLGQCNIAGWILGNPECESLSTARSWSYIVETPNPDNGTCYPGNFINYEELRE

R11241/2022 LRGVAPLHLGQCNIAGWILGNPECESLSTARSWSYIVETPNSDNGTCYPGNFINYEELRE

R10318/2022 LRGIAPLHLGQCNIAGWILGNPECESLSTARSWSYIVETSNSDNGTCYPGDFINYEELRE

R10420/2022 LRGVPPLHLGQCNIAGWILGNPECESLSTARSWSYIVETSNSDNGTCYPGDFINYEELRE

R663/2023 LRGVAPLHLGQCNIAGWILGNPECESLSTARSWSYIVETSNSDNGTCYPGDFINYEELRE

R11323/2022 LRGVAPLHLGQCNIAGWILGNPECESLSTARSWSYIVETSNSDNGTCYPGDFINYEELRE

R93/2023 LRGVAPLHLGQCNIAGWILGNPECESLSTARSWSYIVETSNSDNGTCYPGDFINYEELRE

R11296/2022 LRGVAPLHLGQCNIAGWILGNPECESLSTARSWSYIVETSNSDNGTCYPGDFINYEELRE

R11212/2022 LRGVAPLHLGQCNIAGWILGNPECESLSTARSWSYIVETSNSDNGTCYPGDFINYEELRE

R11298/2022 LRGVAPLHLGQCNIAGWILGNPECESLSTARSWSYIVETSNSDNGTCYPGNFINYEELRE

R10356/2022 LRGVAPLHLGQCNIAGWILGNPECESLSTARSWSYIVETSNSDNGTCYPGNFINYEELRE

R10191/2022 LRGVAPLHLGQCNIAGWILGNPECESLSTARSWSYIVETSNSDNGTCYPGNFINYEELRE

R10316/2022 LRGVAPLHLGQCNIAGWILGNPECESLSTARSWSYIVETSNSDNGTCYPGNFINYEELRE

R11181/2022 LRGVAPLHLGQCNIAGWILGNPECESLSTARSWSYIVETSNSDNGTCYPGNFINYEELRE

R11072/2022 LRGVAPLHLGQCNIAGWILGNPECESLSTARSWSYIVETSNSDNGTCYPGNFINYEELRE

***:.*****:****************************.*.********:*********

A/Wisconsin/588/2019 QLSSVSSFERFEIFPKTSSWPNHDSDNGVTAACPHAGAKSFYKNLIWLVKKGKSYPKINQ

R12781/2022 QLSSVSSFERFEIFPKTSSWPNHDSDNGVTAACSHAGARSFYKNLIWLVKKGKSYPKINQ

R9051/2022 QLSSVSSFERFEIFPKTSSWPNHDSDNGVTAACSHAGARSFYKNLIWLVKKGKSYPKINQ

R11266/2022 QLSSVSSFERFEIFPKTSSWPNHDSDNGVTAACSHAGARSFYKNLIWLVKKGKSYPKINQ

R10300/2022 QLSSVSSFERFEIFPKTSSWPNHDSDNGVTAACSHAGARSFYKNLIWLVKKGKSYPKINQ

R9196/2022 QLSSVSSFERFEIFPKTSSWPNHDSDNGVTAACSHAGARSFYKNLIWLVKKGKSYPKINQ

R10329/2022 QLSSVSSFERFEIFPKTSSWPNHDSDNGVTAACSHAGARSFYKNLIWLVKKGKSYPKINQ

R25/2023 QLSSVSSFERFEIFPKTSSWPNHDSDNGVTAACSHAGARSFYKNLIWLVKKGKSYPKINQ

R53/2023 QLSSVSSFERFEIFPKTSSWPNHDSDNGVTAACSHAGARSFYKNLIWLVKKGKSYPKINQ

R10810/2022 QLSSVSSFERFEIFPKTSSWPNHDSDXGITAACPHAGAKSFYKNLIWLVKKGKSYPKINQ

R463/2023 QLSSVSSFERFEIFPKTSSWPNHDSDNGITAACPHAGAKSFYKNLIWLVKKGKSYPKINQ

R13228/2022 QLSSVSSFERFEIFPKTSSWPNHDSDNGITAACPHAGAKSFYKNLIWLVKKGKSYPKINQ

R49/2023 QLSSVSSFERFEIFPKTSSWPNHDSDNGITAACPHAGAKSFYKNLIWLVKKGKSYPKINQ

R11186/2022 QLSSVSSFERFEIFPKTSSWPNHDSDKGITAACPHAGAKSFYKNLIWLVKKGKSYPKINQ

R10809/2022 QLSSVSSFERFEIFPKTSSWPNHDSDNGITAACPHAGAKSFYKNLIWLVKKGKSYPKINQ

R11241/2022 QLSSVSSFERFEIFPKTSSWPNHDSDNGVTAACPHAGTKSFYKNLIWLVKKGKSYPKINQ

R10318/2022 QLSSVSSFEKFEIFPKTSSWPNHDSDNGVTAACPHAGAKSFYKNLIWLVKKGKSYPKINQ

R10420/2022 QLSSVSSFERFEIFPKTSSWPNHDSDNGVTAACPHAGAKSFYKNLIWLVKKGKSYPKINQ

R663/2023 QLSSVSSFERFEIFPKTSSWPNHDSDNGVTAACPHAGAKSFYKNLIWLVKKGKSYPKINQ

R11323/2022 QLSSVSSFERFEIFPKTSSWPNHDSDNGVTAACPHAGAKSFYKNLIWLVKKGKSYPKINQ

R93/2023 QLSSVSSFERFEIFPKTSSWPNHDSDNGVTAACPHAGAKSFYKNLIWLVKKGKSYPKINQ

R11296/2022 QLSSVSSFERFEIFPKTSSWPNHDSDNGVTAACPHAGAKSFYKNLIWLVKKGKSYPKINQ

R11212/2022 QLSSVSSFERFEIFPKTSSWPNHDSDNGVTAACPHAGAKSFYKNLIWLVKKGKSYPKINQ

R11298/2022 QLSSVSSFERFEIFPKTSSWPNHDSDNGVTAACPHAGAKSFYKNLIWLVKKGKSYPKINQ

R10356/2022 QLSSVSSFERFEIFPKTSSWPNHDSDNGVTAACPHAGAKSFYKNLIWLVKKGKSYPKINQ

R10191/2022 QLSSVSSFERFEIFPKTSSWPNHDSDNGVTAACPHAGAKSFYKNLIWLVKKGKSYPKINQ

R10316/2022 QLSSVSSFERFEIFPKTSSWPNHDSDNGVTAACPHAGAKSFYKNLIWLVKKGKSYPKINQ

R11181/2022 QLSSVSSFERFEIFPKTSSWPNHDSDNGVTAACPHAGAKSFYKNLIWLVKKGKSYPKINQ

R11072/2022 QLSSVSSFERFEIFPKTSSWPNHDSDNGVTAACPHAGAKSFYKNLIWLVKKGKSYPKINQ

*********.**************** *:****.***:.*********************

A/Wisconsin/588/2019 TYINDKGKEVLVLWGIHHPPTIADQQSLYQNADAYVFVGTSRYSKKFKPEIATRPKVRDQ

R12781/2022 TYINDKGKEVLVLWGIHHPPTITDQESLYQNADAYVFVGTSRYSKKFKPEIATRPKVRDQ

R9051/2022 TYINDKGKEVLVLWGIHHPPTITDQESLYQNADAYVFVGTSRYSKKFKPEIATRPKVRDQ

R11266/2022 TYINDKGKEVLVLWGIHHPPTITDQESLYQNADAYVFVGTSRYSKKFKPEIATRPKVRDQ

R10300/2022 TYINDKGKEVLVLWGIHHPPTITDQESLYQNADAYVFVGTSRYSKKFKPEIAARPKVRDQ

R9196/2022 TYINDKGKEVLVLWGIHHPPTITDQESLYQNADAYVFVGTSRYSKKFKPEIAARPKVRDQ

R10329/2022 TYINDKGKEVLVLWGIHHPPTITDQESLYQNADAYVFVGTSRYSKKFKPEIAARPKVRDQ

R25/2023 TYINDKGKEVLVLWGIHHPPTITDQESLYQNADAYVFVGTSRYSKKFKPEIAARPKVRDQ

R53/2023 TYINDKGKEVLVLWGIHHPPTITDQESLYQNADAYVFVGTSRYSKKFKPEIAARPKVRDQ

R10810/2022 TYINDKGKEVLVLWGIHHPPTITDQESLYQNADAYVFVGTSRYSKKFKPEIAARPKVRDQ

R463/2023 TYINDKGKEVLVLWGIHHPPTITDQESLYQNADAYVFVGTSRYSKKFKPEIAARPKVRDQ

R13228/2022 TYINDKGKEVLVLWGIHHPPTITDQESLYQNADAYVFVGTSRYSKKFKPEIAARPKVRDQ

R49/2023 TYINDKGKEVLVLWGIHHPPTITDQESLYQNADAYVFVGTSRYSKKFKPEIAARPKVRDQ

R11186/2022 TYINDKGKEVLVLWGIHHPPTITDQESLYQNADAYVFVGTSRYSKKFKPEIAARPKVRDQ

R10809/2022 TYINDKGKEVLVLWGIHHPPTITDQESLYQNADAYVFVGTSRYSKKFKPEIAARPKVRDQ

R11241/2022 TYINDKGKEVLVLWGIHHPPTITDQESLYQNADAYVFVGTSRYSKKFKPEIAARPKVRDQ

R10318/2022 TYINDKGKEVLVLWGIHHPPTITDQESLYQNADAYVFVGTSRYSKKFKPEIATRPKVRDQ

R10420/2022 TYINDKGKEVLVLWGIHHPPTITDQESLYQNADAYVFVGTSRYSKKFKPEIATRPKVRDQ

R663/2023 TYINDKGKEVLVLWGIHHPPTITDQESLYQNADAYVFVGTSRYSKKFKPEIATRPKVRDQ

R11323/2022 TYINDKGKEVLVLWGIHHPPTITDQESLYQNADAYVFVGTSRYSKKFKPEIATRPKVRDQ

R93/2023 TYINDKGKEVLVLWGIHHPPTITDQESLYQNADAYVFVGTSRYSKKFKPEIATRPKVRDQ

R11296/2022 TYINDKGKEVLVLWGIHHPPTITDQESLYQNADAYVFVGTSRYSKKFKPEIATRPKVRDQ

R11212/2022 TYINDKGKEVLVLWGIHHPPTITDQESLYQNADAYVFVGTSRYSKKFKPEIATRPKVRDQ

R11298/2022 TYINDKGKEVLVLWGIHHPPTITDQESLYQNADAYVFVGTSRYSKKFKPEIAARPKVRDQ

R10356/2022 TYINDKGKEVLVLWGIHHPPTITDQESLYQNADAYVFVGTSRYSKKFKPEIAARPKVRDQ

R10191/2022 TYINDKGKEVLVLWGIHHPPTITDQESLYQNADAYVFVGTSRYSKKFKPEIAARPKVRDQ

R10316/2022 TYINDKGKEVLVLWGIHHPPTITDQESLYQNADAYVFVGTSRYSKKFKPEIAARPKVRDQ

R11181/2022 TYINDKGKEVLVLWGIHHPPTITDQESLYQNADAYVFVGTSRYSKKFKPEIAARPKVRDQ

R11072/2022 TYINDKGKEVLVLWGIHHPPTITDQESLYQNADAYVFVGTSRYSKKFKPEIAARPKVRDQ

**********************:**:**************************:*******

A/Wisconsin/588/2019 EGRMNYYWTLVEPGDKITFEATGNLVAPRYAFTMERDAGSGIIISDTPVHDCNTTCQTPE

R12781/2022 AGRMNYYWTLVEPGDKITFEATGNLVAPRYAFTMEKEAGSGIIISDTPVHDCNATCQTPE

R9051/2022 AGRMNYYWTLVEPGDKITFEATGNLVAPRYAFTMEKEAGSGIIISDTPVHDCNATCQTPE

R11266/2022 AGRMNYYWTLVEPGDKITFEATGNLVAPRYAFTMEKEAGSGIIISDTPVHDCNATCQTPE

R10300/2022 AGRMNYYWTLVEPGDKITFEATGNLVAPRYAFTMENEAGSGIIISDTPVHNCNATCQTPE

R9196/2022 AGRMKYYWTLVEPGDKITFEATGNLVAPRYAFTMEKEAGSGIIISDTPVHNCNATCQTPE

R10329/2022 AGRMNYYWTLVEPGDKITFEATGNLVAPRYAFTMEKEAGSGIIISDTPVHNCNATCQTPE

R25/2023 AGRMNYYWTLVEPGDKITFEATGNLVAPRYAFTMEKEAGSGIIISDTPVHNCNATCQTPE

R53/2023 AGRMNYYWTLVEPGDKITFEATGNLVAPRYAFTMEKEAGSGIIISDTPVHNCNATCQTPE

R10810/2022 AGRMNYYWTLVEPGDKITFEATGNLVAPRYAFTMEKDAGSGIIISDXPVQDCNTTCQTPE

R463/2023 AGRMNYYWTLVEPGDKITFEATGNLVAPRYAFTMEKDAGSGIIISDTPVQDCNTTCQTPE

R13228/2022 AGRMNYYWTLVEPGDKITFEATGNLVAPRYAFTMEKDAGSGIIISDTPVQDCNTTCQTPE

R49/2023 AGRMNYYWTLVEPGDKITFEATGNLVAPRYAFTMEKDAGSGIIISDTPVQDCNTTCQTPE

R11186/2022 AGRMNYYWTLVEPGDKITFEATGNLVAPRYAFTMEKDAGSGIIISDTPVQDCNTTCQTPE

R10809/2022 AGRMNYYWTLVEPGDKITFEATGNLVAPRYAFTMEKDAGSGIIISDTPVQDCNTTCQTPE

R11241/2022 AGRMNYYWTLVEPGDKITFEATGNLVAPRYAFTMEKDAGSGIIISDTPVHDCNATCQTPE

R10318/2022 AGRMNYYWTLVEPGDKITFEATGNLVAPRYAFTMENDAGSGIIISNTPVHDCNTTCQTPE

R10420/2022 AGRMNYYWTLVEPGDKITFEATGNLVAPRYAFTMEKDAGSGIIISDTPVHDCNTTCQTPE

R663/2023 AGRMNYYWTLVEPGDKITFEATGNLVAPRYAFTMEKDAGSGIIISDTPVHDCNTTCQTPE

R11323/2022 AGRMNYYWTLVEPGDKITFEATGNLVAPRYAFTMEKDAGSGIIISDTPVHDCNTTCQTPE

R93/2023 AGRMNYYWTLVEPGDKITFEATGNLVAPRYAFTMEKDAGSGIIISDTPVHDCNTTCQTPE

R11296/2022 AGRMNYYWTLVEPGDKITFEATGNLVAPRYAFTMEKDAGSGIIISDTPVHDCNTTCQTPE

R11212/2022 AGRMNYYWTLVEPGDKITFEATGNLVAPRYAFTMEKDAGSGIIISDTPVHDCNTTCQTPE

R11298/2022 AGRMNYYWTLVEPGDKITFEATGNLVAPRYAFTMEKDAGSGIIISDTPVHDCNTTCQTPE

R10356/2022 AGRMNYYWTLVEPGDKITFEATGNLVAPRYAFTMEKDAGSGIIISDTPVHDCNTTCQTPE

R10191/2022 AGRMNYYWTLVEPGDKITFEATGNLVAPRYAFTMEKDAGSGIIISDTPVHDCNTTCQTPE

R10316/2022 AGRMNYYWTLVEPGDKITFEATGNLVAPRYAFTMENDAGSGIIISDTPVHDCNTTCQTPE

R11181/2022 AGRMNYYWTLVEPGDKITFEATGNLVAPRYAFTMEKDAGSGIIISDTPVHDCNTTCQTPE

R11072/2022 AGRMNYYWTLVEPGDKITFEATGNLVAPRYAFTMEKDAGSGIIISDTPVHDCNTTCQTPE

***:******************************.:********: **::**:******

A/Wisconsin/588/2019 GAINTSLPFQNVHPITIGKCPKYVKSTKLRLATGLRNVPSIQSRGLFGAIAGFIEGGWTG

R12781/2022 GAINTSLPFQNVHPITIGKCPKYVRSTKLRLATGLRNVPSIQSRGLFGAIAGFIEGGWTG

R9051/2022 GAINTSLPFQNVHPITIGKCPKYVRSTKLRLATGLRNVPSIQSRGLFGAIAGFIEGGWTG

R11266/2022 GAINTSLPFQNVHPITIGKCPKYVRSTKLRLATGLRNVPSIQSRGLFGAIAGFIEGGWTG

R10300/2022 GAINTSLPFQNVHPITIGKCPKYVRSTKLRLATGLRNVPSIQSRGLFGAIAGFIEGGWTG

R9196/2022 GAINTSLPFQNVHPITIGKCPKYVRSTKLRLATGLRNVPSIQSRGLFGAIAGFIEGGWTG

R10329/2022 GAINTSLPFQNVHPITIGKCPKYVRSTKLRLATGLRNVPSIQSRGLFGAIAGFIEGGWTG

R25/2023 GAINTSLPFQNVHPITIGKCPKYVRSTKLRLATGLRNVPSIQSRGLFGAIAGFIEGGWTG

R53/2023 GAINTSLPFQNVHPITIGKCPKYVRSTKLRLATGLRNVPSIQSRGLFGAIAGFIEGGWTG

R10810/2022 GAINTSLPFQNVHPITIGKCPKYVRSTKLRLATGLRNIPSIQSRGLFGAIAGFIEGGWTG

R463/2023 GAINTSLPFQNVHPITIGKCPKYVRSTKLRLATGLRNIPSIQSRGLFGAIAGFIEGGWTG

R13228/2022 GAINTSLPFQNVHPITIGKCPKYVRSTKLRLATGLRNIPSIQSRGLXGAIAGFIEGGWTG

R49/2023 GAINTSLPFQNVHPITIGKCPKYVRSTKLRLATGLRNIPSIQSRGLFGAIAGFIEGGWTG

R11186/2022 GAINTSLPFQNVHPITIGKCPKYVRSTKLRLATGLRNIPSIQSRGLFGAIAGFIEGGWTG

R10809/2022 GAINTSLPFQNVHPITIGKCPKYVRSTKLRLATGLRNIPSIQSRGLFGAIAGFIEGGWTG

R11241/2022 GAINTSLPFQNVHPITIGKCPKYVRSTKLRLATGLRNIPSIQSRGLFGAIAGFIEGGWTG

R10318/2022 GAINTSLPFQNVHPITIGKCPKYVRSTKLRLATGLRNVPSIQSRGLFGAIAGFIEGGWTG

R10420/2022 GAINTSLPFQNVHPITIGKCPKYVRSTKLRLATGLRNVPSIQSRGLFGAIAGFIEGGWTG

R663/2023 GAINTSLPFQNVHPITIGKCPKYVRSTKLRLATGLRNVPSIQSRGLFGAIAGFIEGGWTG

R11323/2022 GAINTSLPFQNVHPITIGKCPKYVRSTKLRLATGLRNVPSIQSRGLFGAIAGFIEGGWTG

R93/2023 GAINTSLPFQNVHPITIGKCPKYVRSTKLRLATGLRNVPSIQSRGLFGAIAGFIEGGWTG

R11296/2022 GAINTSLPFQNVHPITIGKCPKYVRSTKLRLATGLRNVPSIQSRGLFGAIAGFIEGGWTG

R11212/2022 GAINTSLPFQNVHPITIGKCPKYVRSTKLRLATGLRNVPSIQSRGLFGAIAGFIEGGWTG

R11298/2022 GAINTSLPFQNVHPITIGKCPKYVRSTKLRLATGLRNVPSIQSRGLFGAIAGFIEGGWTG

R10356/2022 GAINTSLPFQNVHPITIGKCPKYVRSTKLRLATGLRNVPSIQSRGLFGAIAGFIEGGWTG

R10191/2022 GAINTSLPFQNVHPITIGKCPKYVRSTKLRLATGLRNVPSIQSRGLFGAIAGFIEGGWTG

R10316/2022 GAINTSLPFQNVHPITIGKCPKYVRSTKLRLATGLRNVPSIQSRGLFGAIAGFIEGGWTG

R11181/2022 GAINTSLPFQNVHPITIGKCPKYVRSTKLRLATGLRNVPSIQSRGLFGAIAGFIEGGWTG

R11072/2022 GAINTSLPFQNVHPITIGKCPKYVRSTKLRLATGLRNVPSIQSRGLFGAIAGFIEGGWTG

************************.************:******** *************

A/Wisconsin/588/2019 MVDGWYGYHHQNEQGSGYAADLKSTQNAIDKITNKVNSVIEKMNTQFTAVGKEFNHLEKR

R12781/2022 MVDGWYGYHHQNDQGSGYAADLKSTQNAIDKITNKVNSVIEKMNTQFTAVGKEFNHLEKR

R9051/2022 MVDGWYGYHHQNDQGSGYAADLKSTQNAIDKITNKVNSVIEKMNTQFTAVGKEFNHLEKR

R11266/2022 MVDGWYGYHHQNDQGSGYAADLKSTQNAIDKITNKVNSVIEKMNTQFTAVGKEFNHLEKR

R10300/2022 MVDGWYGYHHQNDQGSGYAADLKSTQNAIDKITNKVNSVIEKMNTQFTAVGKEFNHLEKR

R9196/2022 MVDGWYGYHHQNDQGSGYAADLKSTQNAIDKITNKVNSVIEKMNTQFTAVGKEFNHLEKR

R10329/2022 MVDGWYGYHHQNDQGSGYAADLKSTQNAIDKITNKVNSVIEKMNTQFTAVGKEFNHLEKR

R25/2023 MVDGWYGYHHQNDQGSGYAADLKSTQNAIDKITNKVNSVIEKMNTQFTAVGKEFNHLEKR

R53/2023 MVDGWYGYHHQNDQGSGYAADLKSTQNAIDKITNKVNSVIEKMNTQFTAVGKEFNHLEKR

R10810/2022 MVDGWYGYHHQNEQGSGYAADLKSTQNAIDKITNKVNSVIEKMNTQFTAVGKEFNHLEKR

R463/2023 MVDGWYGYHHQNEQGSGYAADLKSTQNAIDKITNKVNSVIEKMNTQFTAVGKEFNHLEKR

R13228/2022 MVDGWYGYHHQNEQGSGYAADLKSTQNAIDKITNKVNSVIEKMNTQFTAVGKEFNHLEKR

R49/2023 MVDGWYGYHHQNEQGSGYAADLKSTQNAIDKITNKVNSVIEKMNTQFTAVGKEFNHLEKR

R11186/2022 MVDGWYGYHHQNEQGSGYAADLKSTQNAIDKITNKVNSVIEKMNTQFTAVGKEFNHLEKR

R10809/2022 MVDGWYGYHHQNEQGSGYAADLKSTQNAIDKITNKVNSVIEKMNTQFTAVGKEFNHLEKR

R11241/2022 MVDGWYGYHHQNEQGSGYAADLKSTQNAIDKITNKVNSVIEKMNTQFTAVGKEFNHLEKR

R10318/2022 MVDGWYGYHHQNEQGSGYAADLKSTQNAIDKITNKVNSVIEKMNTQFTAVGKEFNHLEKR

R10420/2022 MVDGWYGYHHQNEQGSGYAADLKSTQNAIDKITNKVNSVIEKMNTQFTAVGKEFNHLEKR

R663/2023 MVDGWYGYHHQNEQGSGYAADLKSTQNAIDKITNKVNSVIEKMNTQFTAVGKEFNHLEKR

R11323/2022 MVDGWYGYHHQNEQGSGYAADLKSTQNAIDKITNKVNSVIEKMNTQFTAVGKEFNHLEKR

R93/2023 MVDGWYGYHHQNEQGSGYAADLKSTQNAIDKITNKVNSVIEKMNTQFTAVGKEFNHLEKR

R11296/2022 MVDGWYGYHHQNEQGSGYAADLKSTQNAIDKITNKVNSVIEKMNTQFTAVGKEFNHLEKR

R11212/2022 MVDGWYGYHHQNEQGSGYAADLKSTQNAIDKITNKVNSVIEKMNTQFTAVGKEFNHLEKR

R11298/2022 MVDGWYGYHHQNEQGSGYAADLKSTQNAIDKITNKVNSVIEKMNTQFTAVGKEFNHLEKR

R10356/2022 MVDGWYGYHHQNEQGSGYAADLKSTQNAIDKITNKVNSVIEKMNTQFTAVGKEFNHLEKR

R10191/2022 MVDGWYGYHHQNEQGSGYAADLKSTQNAIDKITNKVNSVIEKMNTQFTAVGKEFNHLEKR

R10316/2022 MVDGWYGYHHQNEQGSGYAADLKSTQNAIDKITNKVNSVIEKMNTQFTAVGKEFNHLEKR

R11181/2022 MVDGWYGYHHQNEQGSGYAADLKSTQNAIDKITNKVNSVIEKMNTQFTAVGKEFNHLEKR

R11072/2022 MVDGWYGYHHQNEQGSGYAADLKSTQNAIDKITNKVNSVIEKMNTQFTAVGKEFNHLEKR

************:***********************************************

A/Wisconsin/588/2019 IENLNKKVDDGFLDIWTYNAELLVLLENERTLDYHDSNVKNLYEKVRNQLKNNAKEIGNG

R12781/2022 IENLNKKVDDGFLDVWTYNAELLVLLENERTLDYHDSNVKNLYEKVRHQLKNNAKEIGNG

R9051/2022 IENLNKKVDDGFLDVWTYNAELLVLLENERTLDYHDSNVKNLYEKVRHQLKNNAKEIGNG

R11266/2022 IENLNKKVDDGFLDVWTYNAELLVLLENERTLDYHDSNVKNLYEKVRHQLKNNAKEIGNG

R10300/2022 IENLNKKVDDGFLDVWTYNAELLVLLENERTLDYHDSNVKNLYEKVRHQLKNNAKEIGNG

R9196/2022 IENLNKKVDDGFLDVWTYNAELLVLLENERTLDYHDSNVKNLYEKVRHQLKNNAKEIGNG

R10329/2022 IENLNKKVDDGFLDVWTYNAELLVLLENERTLDYHDSNVKNLYEKVRHQLKNNAKEIGNG

R25/2023 IENLNKKVDDGFLDVWTYNAELLVLLENERTLDYHDSNVKNLYEKVRHQLKNNAKEIGNG

R53/2023 IENLNKKVDDGFLDVWTYNAELLVLLENERTLDYHDSNVKNLYEKVRHQLKNNAKEIGNG

R10810/2022 IENLNKKVDDGFLDIWTYNAELLVLLENERTLDYHDSNVKNLYEKVRNQLKNNAKEIGNG

R463/2023 IENLNKKVXDGFLDIWTYNAELLVLLENERTLDYHDSNVKNLYEKVRNQLKNNAKEIGNG

R13228/2022 IENLNKKVDDGFLDIWTYNAELLVLLENERTLDYHDSNVKNLYEKVRNQLKNNAKEIGNG

R49/2023 IENLNKKVDDGFLDIWTYNAELLVLLENERTLDYHDSNVKNLYEKVRNQLKNNAKEIGNG

R11186/2022 IENLNKKVDDGFLDIWTYNAELLVLLENERTLDYHDSNVKNLYEKVRNQLKNNAKEIGNG

R10809/2022 IENLNKKVDDGFLDIWTYNAELLVLLENERTLDYHDSNVKNLYEKVRNQLKNNAKEIGNG

R11241/2022 IENLNKKVDDGFLDIWTYNAELLVLLENERTLDYHDSNVKNLYEKVRNQLKNNAKEIGNG

R10318/2022 IENLNKKVDDGFLDVWTYNAELLVLLENERTLDYHDSNVKNLYEKVRNQLKNNAKEIGNG

R10420/2022 IENLNKKVDDGFLDIWTYNAELLVLLENERTLDYHDSNVKNLYEKVRNQLKNNAKEIGNG

R663/2023 IENLNKKVDDGFLDVWTYNAELLVLLENERTLDYHDSNVKNLYEKVRNQLKNNAKEIGNG

R11323/2022 IENLNKKVDDGFLDVWTYNAELLVLLENERTLDYHDSNVKNLYEKVRNQLKNNAKEIGNG

R93/2023 IENLNKKVDDGFLDVWTYNAELLVLLENERTLDYHDSNVKNLYEKVRNQLKNNAKEIGNG

R11296/2022 IENLNKKVDDGFLDVWTYNAELLVLLENERTLDYHDSNVKNLYEKVRNQLKNNAKEIGNG

R11212/2022 IENLNKKVDDGFLDIWTYNAELLVLLENERTLDYHDSNVKNLYEKVRNQLKNNAKEIGNG

R11298/2022 VENLNKKVDDGFLDIWTYNAELLVLLENERTLDYHDSNVKNLYEKVRNQLKNNAKEIGNG

R10356/2022 VENLNKKVDDGFLDIWTYNAELLVLLENERTLDYHDSNVKNLYEKVRNQLKNNAKEIGNG

R10191/2022 IENLNKKVDDGFLDIWTYNAELLVLLENERTLDYHDSNVKNLYEKVRNQLKNNAKEIGNG

R10316/2022 IENLNKKVDDGFLDIWTYNAELLVLLENERTLDYHDSNVKNLYEKVRNQLKNNAKEIGNG

R11181/2022 IENLNKKVDDGFLDIWTYNAELLVLLENERTLDYHDSNVKNLYEKVRNQLKNNAKEIGNG

R11072/2022 IENLNKKVDDGFLDIWTYNAELLVLLENERTLDYHDSNVKNLYEKVRNQLKNNAKEIGNG

:******* *****:********************************:************

A/Wisconsin/588/2019 CFEFYHKCDNTCMESVKNGTYDYPKYSEEAKLNREKIDGVKLDSTRIYQILAIYSTVASS

R12781/2022 CFEFYHKCDNTCMESVKNGTYDYPKYSEEAKLNREKIDGVKLDSTRIYQILAIYSTVASS

R9051/2022 CFEFYHKCDNTCMESVKNGTYDYPKYSEEAKLNREKIDGVKLDSTRIYQILAIYSTVASS

R11266/2022 CFEFYHKCDNTCMESVKNGTYDYPKYSEEAKLNREKIDGVKLDSTRIYQILAIYSTVASS

R10300/2022 CFEFYHKCDNTCMESVKNGTYDYPKYSEEAKLNREKIDGVKLDSTRIYQILAIYSTVASS

R9196/2022 CFEFYHKCDNTCMESVKNGTYDYPKYSEEAKLNREKIDGVKLDSTRIYQILAIYSTVASS

R10329/2022 CFEFYHKCDNTCMESVKNGTYDYPKYSEEAKLNREKIDGVKLDSTRIYQILAIYSTVASS

R25/2023 CFEFYHKCDNTCMESVKNGTYDYPKYSEEAKLNREKIDGVKLDSTRIYQILAIYSTVASS

R53/2023 CFEFYHKCDNTCMESVKNGTYDYPKYSEEAKLNREKIDGVKLDSTRIYQILAIYSTVASS

R10810/2022 CFEFYHKCDNTCMESVKNGTYDYPKYSEEAKLNREKIDGVKLDSTRIYQILAIYSTVASS

R463/2023 CFEFYHKCDNTCMESVKNGTYDYPKYSEEAKLNREKIDGVKLDSTRIYQILAIYSTVASS

R13228/2022 CFEFYHKCDNTCMESVKNGTYDYPKYSEEAKLNREKIDGVKLDSTRIYQILAIYSTVASS

R49/2023 CFEFYHKCDNTCMESVKNGTYDYPKYSGEAKLNREKIDGVKLDSTRIYQILAIYSTVASS

R11186/2022 CFEFYHKCDNTCMESVKNGTYDYPKYSEEAKLNREKIDGVKLDSTRIYQILAIYSTVASS

R10809/2022 CFEFYHKCDNTCMESVKNGTYDYPKYSEEAKLNREKIDGVKLDSTRIYQILAIYSTVASS

R11241/2022 CFEFYHKCDNTCMESVKNGTYDYPKYSEEAKLNREKIDGVKLDSTRIYQILAIYSTVASS

R10318/2022 CFEFYHKCDNTCMESVKNGTYDYPKYSEEAKLNREKIDGVKLDSTRIYQILAIYSTVASS

R10420/2022 CFEFYHKCDNTCMESVKNGTYDYPKYSEEAKLNREKIDGVKLDSTRIYQILAIYSTVASS

R663/2023 CFEFYHKCDNTCMESVKNGTYDYPKYSEEAKLNREKIDGVKLDSTRIYQILAIYSTVASS

R11323/2022 CFEFYHKCDNTCMESVKNGTYDYPKYSEEAKLNREKIDGVKLDSTRIYQILAIYSTVASS

R93/2023 CFEFYHKCDNTCMESVKNGTYDYPKYSEEAKLNREKIDGVKLDSTRIYQILAIYSTVASS

R11296/2022 CFEFYHKCDNTCMESVKNGTYDYPKYSEEAKLNREKIDGVKLDSTRIYQILAIYSTVASS

R11212/2022 CFEFYHKCDNTCMESVKNGTYDYPKYSEEAKLNREKIDGVKLDSTRIYQILAIYSTVASS

R11298/2022 CFEFYHKCDNTCMESVKNGTYDYPKYSEEAKLNREKIDGVKLDSTRIYQILAIYSTVASS

R10356/2022 CFEFYHKCDNTCMESVKNGTYDYPKYSEEAKLNREKIDGVKLDSTRIYQILAIYSTVASS

R10191/2022 CFEFYHKCDNTCMESVKNGTYDYPKYSEEAKLNREKIDGVKLDSTRIYQILAIYSTVASS

R10316/2022 CFEFYHKCDNTCMESVKNGTYDYPKYSEEAKLNREKIDGVKLDSTRIYQILAIYSTVASS

R11181/2022 CFEFYHKCDNTCMESVKNGTYDYPKYSEEAKLNREKIDGVKLDSTRIYQILAIYSTVASS

R11072/2022 CFEFYHKCDNTCMESVKNGTYDYPKYSEEAKLNREKIDGVKLDSTRIYQILAIYSTVASS

*************************** ********************************

A/Wisconsin/588/2019 LVLVVSLGAISFWMCSNGSLQCRICI

R12781/2022 LVLVVSLGAISFWMCSNGSLQCRICI

R9051/2022 LVLVVSLGAISFWMCSNGSLQCRICI

R11266/2022 LVLVVSLGAISFWMCSNGSLQCRICI

R10300/2022 LVLVVSLGAISFWMCSNGSLQCRICI

R9196/2022 LVLVVSLGAISFWMCSNGSLQCRICI

R10329/2022 LVLVVSLGAISFWMCSNGSLQCRICI

R25/2023 LVLVVSLGAISFWMCSNGSLQCRICI

R53/2023 LVLVVSLGAISFWMCSNGSLQCRICI

R10810/2022 LVLVVSLGAISFWMCSNGSLQCRICI

R463/2023 LVLVVSLGAISFWMCSNGSLQCRICI

R13228/2022 LVLVVSLGAISFWMCSNGSLQCRICI

R49/2023 LVLVVSLGAISFWMCSNGSLQCRICI

R11186/2022 LVLVVSLGAISFWMCSNGSLQCRICI

R10809/2022 LVLVVSLGAISFWMCSNGSLQCRICI

R11241/2022 LVLVVSLGAISFWMCSNGSLQCRICI

R10318/2022 LVLVVSLGAISFWMCSNGSLQCRICI

R10420/2022 LVLVVSLGAISFWMCSNGSLQCRICI

R663/2023 LVLVVSLGAISFWMCSNGSLQCRICI

R11323/2022 LVLVVSLGAISFWMCSNGSLQCRICI

R93/2023 LVLVVSLGAISFWMCSNGSLQCRICI

R11296/2022 LVLVVSLGAISFWMCSNGSLQCRICI

R11212/2022 LVLVVSLGAISFWMCSNGSLQCRICI

R11298/2022 LVLVVSLGAISFWMCSNGSLQCRICI

R10356/2022 LVLVVSLGAISFWMCSNGSLQCRICI

R10191/2022 LVLVVSLGAISFWMCSNGSLQCRICI

R10316/2022 LVLVVSLGAISFWMCSNGSLQCRICI

R11181/2022 LVLVVSLGAISFWMCSNGSLQCRICI

R11072/2022 LVLVVSLGAISFWMCSNGSLQCRICI

**************************
